# Scale-Free Analysis of Intraoperative ECoG During Awake Craniotomy for Glioma

**DOI:** 10.3389/fonc.2020.625474

**Published:** 2021-02-23

**Authors:** Diana Cristina Ghinda, Ben Lambert, Junfeng Lu, Ning Jiang, Eve Tsai, Adam Sachs, Jin-Song Wu, Georg Northoff

**Affiliations:** ^1^ Department of Neurosurgery, The Ottawa Hospital, University of Ottawa, Ottawa Hospital Research Institute, Ottawa, ON, Canada; ^2^ Glioma Surgery Division, Department of Neurosurgery, Huashan Hospital, Shanghai Medical College, Fudan University, Shanghai, China; ^3^ Mind, Brain Imaging and Neuroethics, Institute of Mental Health Research, University of Ottawa, Ottawa, ON, Canada; ^4^ Faculty of Engineering, Department of Systems Design Engineering, University of Waterloo, Waterloo, ON, Canada

**Keywords:** glioma, electrocorticography, brain tumor, awake craniotomy, glioma-related epilepsy, scale-free measures

## Abstract

**Background:**

Electrocorticography (ECoG) has been utilized in many epilepsy cases however, the use of this technique for evaluating electrophysiological changes within tumoral zones is spare. Nonetheless, epileptic activities seem to arise from the neocortex surrounding the gliomas suggesting a link between epileptogenesis and glioma cell infiltration in the peritumoral area. The purpose of this study was to implement novel scale-free measures to assess how cortical physiology is altered by the presence of an invasive brain tumor.

**Methods:**

Twelve patients undergoing an awake craniotomy for resection of a supratentorial glioma were included. ECoG data over the main tumor and the exposed surroundings was acquired intra-operatively just prior to tumor resection. Six of the patients presented with seizures and had data acquired both in the awake and anesthetic state. The corresponding anatomical location of each electrode in relation to the macroscopically-detectable tumor was recorded using the neuronavigation system based on structural anatomical images obtained pre-operatively. The electrodes were classified into tumoral, healthy or peritumoral based on the macroscopically detectable tumoral tissue from the pre-operative structural MRI.

**Results:**

The electrodes overlying the tumoral tissue revealed higher power law exponent (PLE) values across tumoral area compared to the surrounding tissues. The difference between the awake and anesthetic states was significant in the tumoral and healthy tissue (p < 0.05) but not in the peritumoral tissue. The absence of a significant PLE reduction in the peritumoral tissue from the anesthetic to the awake state could be considered as an index of the presence or absence of infiltration of tumor cells into the peritumoral tissue.

**Conclusions:**

The current study portrays for the first time distinct power law exponent features in the tumoral tissue, which could provide a potential novel electrophysiological marker in the future. The distinct features seen in the peritumoral tissue of gliomas seem to indicate the area where both the onset of epileptiform activity and the tumor infiltration take place.

## Introduction

Although the main presenting symptom of low-grade gliomas are seizures, the pathophysiological mechanisms and related structural-functional abnormalities underlying the epileptogenesis in patients with these tumors remain unclear (1–3).

The recording of electrical changes in the brain by electrodes placed directly on the cerebral cortex, electrocorticography (ECoG), has previously been used for delineation of tumors when the non-invasive imaging techniques were not yet available. Similarly, the electroencephalogram (EEG) had localizing value in cases of brain tumor and epilepsy and the occurrence of delta waves was thought to be a better guide than areas of electrical silence (4). Subcortical tumors have also been localized by the presence of slow wave activity over the tumor while the epileptogenic focus could be determined by focal spontaneous spiking, induction of seizures by electrical stimulation and occurrence of long-lasting after-discharges (5). The use of ECoG for cerebral tumors revealed distinct changes in the setting of infiltrative tumors (6), for instance slow delta-waves were found in the surrounding edema (7). The origin of these slow waves was assumed to be related to intracranial hypertension however, another study analyzing the morphological and pathophysiological features of the peritumoral area concluded that those changes result from direct influence of the tumor on brain parenchyma, rather than from peritumoral edema or intracranial hypertension (8). As such, historically, ECoG was not only of scientific interest but was also of great practical value for neurosurgery.

**Figure 1:**
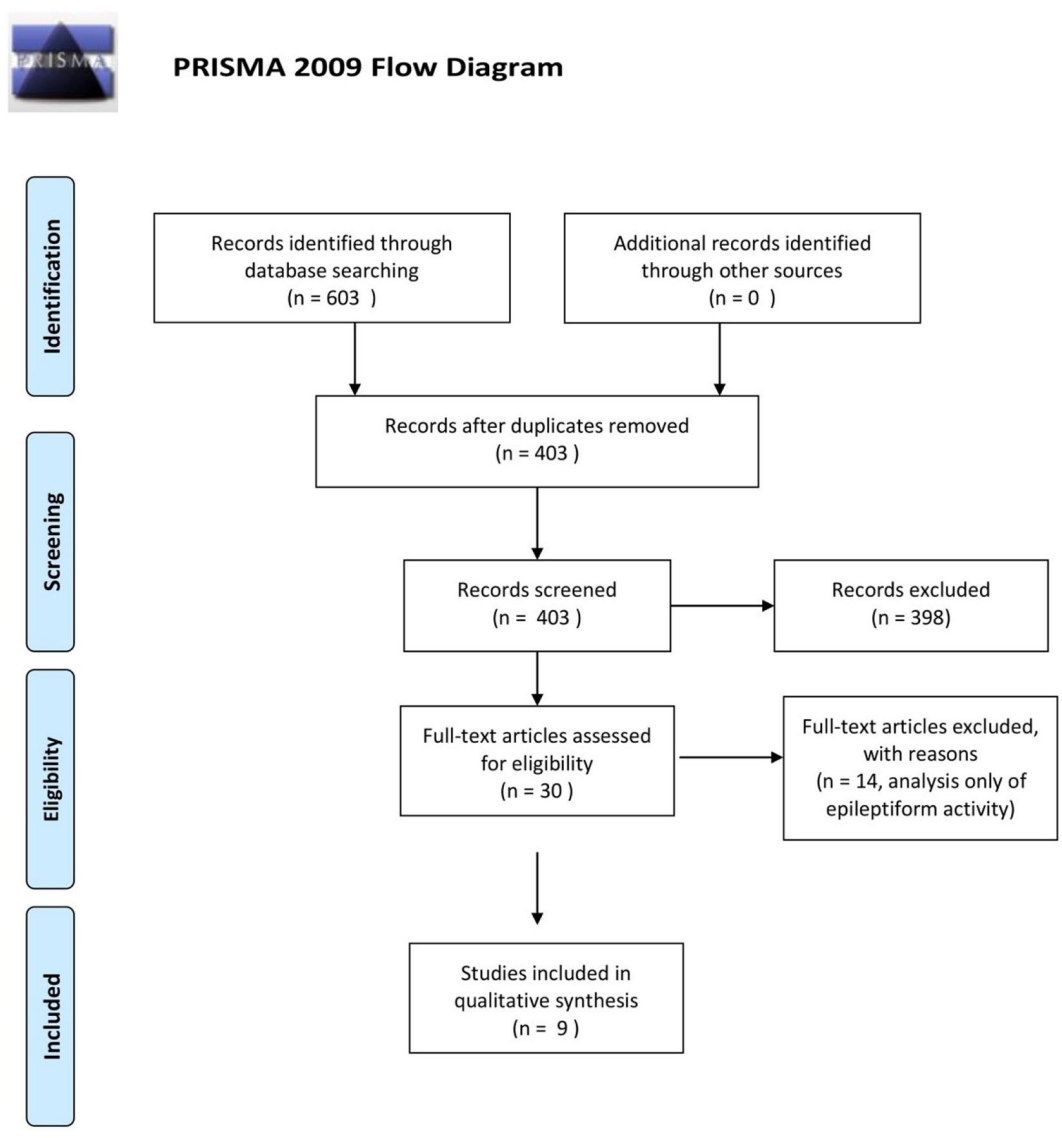
PRISMA flow diagram.

Besides delineating the eloquent areas that need to be preserved during the surgical resection, ECoG use for assessment of electrophysiological changes occurring in the setting of glioma is currently a relatively non-explored domain. It is postulated that the peritumoral neocortex around gliomas represents a pivotal structure both for the genesis of glioma related epilepsy (GRE) and for infiltration by glioma cells, which has significant implications in terms of the treatment recommended to the patients and their oncological prognosis (3, 9–12). In a systematic review performed on this subject, using medical subject headings and text words related to ECoG, epilepsy and gliomas in MEDLINE (OVID interface, 1946 onward), EMBASE (OVID interface, 1947 onward), and the Cochrane Central Register of Controlled Trials, we found only nine articles using EEG or ECoG for assessment of glioma-related electrophysiological changes (**Figure 1**). In this paper, we outline a novel *in vivo* electrophysiological investigation of tumoral tissue in patients undergoing awake craniotomy for tumor resection. Subsequently, we discuss our findings in the context of the available literature.

## Methods

### Subjects and Recordings

Twelve patients with a newly diagnosed intracranial glioma, out of which six presenting with multiple seizures, were enrolled after providing informed consent. Patients were eligible for inclusion if they were aged ≥18 years, presented with a supratentorial lesion suggestive of a glioma, and had an indication for surgery as confirmed by an experienced neurosurgery team (J-S.W., J.L.). Of the patients sequentially admitted for treatment, we excluded patients not eligible for awake surgery, patients with other brain pathologies and those who had undergone previous cranial irradiation or chemotherapy and/or for whom electrophysiological recordings could not be obtained.

The patients’ demographics including the presenting symptoms are presented in **Table 1**. All patients underwent awake craniotomy for tumor resection. A monitored anesthesia care approach was adopted for all patients as per previous publications (13). After the anesthesiologists administered premedication by infusing midazolam (0.02–0.03 mg/kg) and 5 mg of tropisetron, intravenous lines, a central venous catheter, an arterial line, and a urethral catheter were inserted. The supraorbital, supratrochlear, zygomaticotemporal, auriculotemporal, greater occipital, and lesser occipital nerves were blocked bilaterally using a mixture of lidocaine (0.67%) and ropivacaine (0.5%). Once the patient was brought into moderate sedation with boluses of intravenous propofol, the head was fixed into its fitted position using a custom-designed high-field MRI-safe head holder (DORO Radiolucent Headrest System, Pro Med Instruments GmbH, Freiburg, Germany), which was integrated with an intraoperative MR imaging system (IMRIS™). Once the scalp was prepared and draped, remifentanil (0.01–0.03 lg/kg/min) or dexmedetomidine (0.1–0.7 lg/kg/h) were administered for analgesia. To minimize brain swelling, mannitol (1 g/kg) was infused intravenously before the dura opening. Because of the minimal draping used, no laryngeal mask airway or endotracheal tubing was applied.

**Table 1:** Patient’s demographics and clinical data.

Patient #	Age, gender	AED prior to admission	Tumor Location	WHO Grade	IDH 1 status	Presenting Symptoms
1	50, F	None	Frontal	III	Wild-type	Focal aware seizure, non-motor onset-sensory
2	40, M	Oxycarbazepine	Frontal & insular	III	Wild-type	Focal aware seizure, motor onset-epileptic spasm
3	39, M	VPA	Temporal & insular	IV	Wild-type	Focal aware seizure, non-motor onset-autonomic
4	34, M	VPA	Fronto-temporal	II	Mutant	Generalized seizure, motor – tonic-clonic
5	29, F	VPA	Parietal	II	Mutant	Generalized seizure, motor – tonic-clonic
6	35, F	None	Frontal	II	Mutant	Generalized seizure, motor – tonic-clonic
7	52, F	None	Frontal	IV	Wild-type	Contralateral weakness
8	49, F	None	Frontal	II	Mutant	Incidental
9	42, M	None	Frontal	II	Mutant	Incidental
10	48, M	None	Frontal	II	Mutant	Contralateral sensory changes
11	33, F	None	Frontal	II	Mutant	Headaches
12	41, F	None	Frontal & parietal	II	Mutant	Headaches

AED, anti-epileptic drugs; IDH, Isocitrate Dehydrogenase; WHO, World Health Organisation; VPA, Valproic Acid.

The ECoG grid was placed over the exposed brain surface as indicated by the needs of the patient. ECoG signals were recorded with a 20, 32 or 48-channel grid of 4.0 mm diameter electrodes with 2.3mm exposure (Ad-tech, Germany) connected to a BP amplifier (Brain Product, Germany). The reference electrode was placed on the contralateral earlobe to avoid any potential increase in coherence due to the reference electrode location.

5 min of recording in the awake state were acquired prior to the tumor resection. For the six patients presenting with seizures, the ECoG signal was also acquired in the anesthetic state. The signals were sampled at 1,000 Hz with a hardware filter between 0.01 and 1,000 Hz. The ECoG electrodes for each patient were classified in TT (tumoral tissue), HT (macroscopically healthy-tissue) and PT (peritumoral tissue) according to the radiological defined boundaries as per the conventional structural MRI.

### ECoG Localization Method *via* Intra-Operative Photography

**Figure 2:**
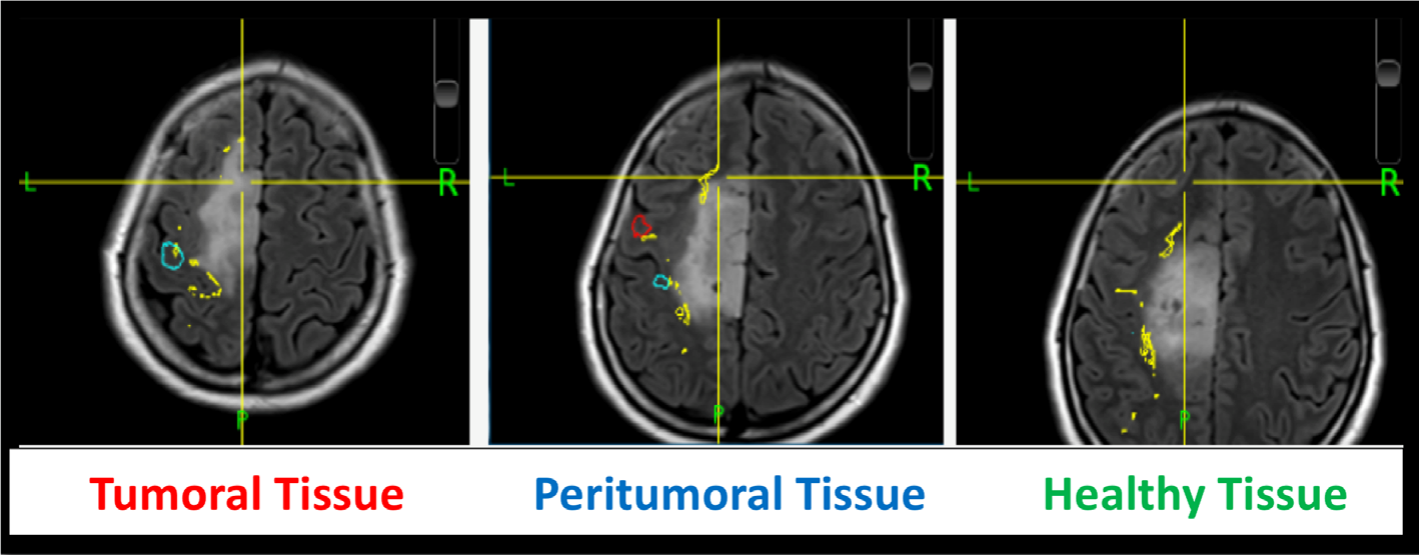
ECoG localization method *via* intra-operative neuronavigation.

**Figure 3:**
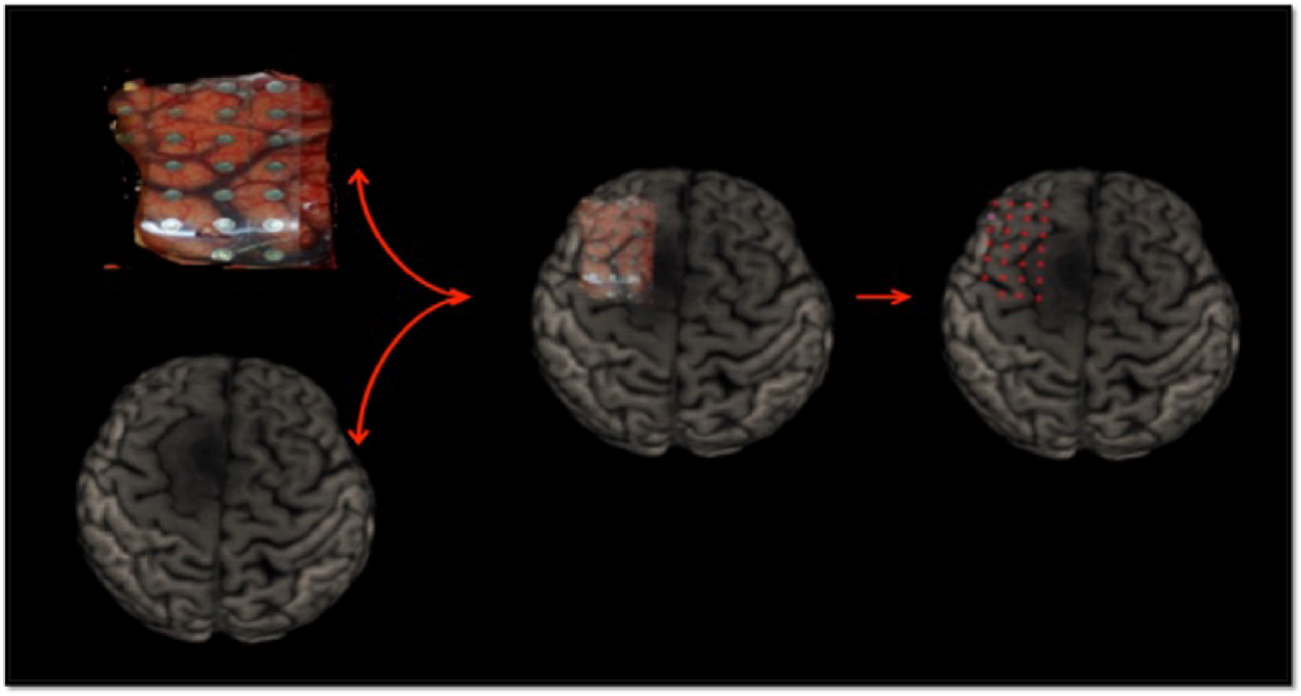
ECoG localization method *via* intra-operative photograph merged with the cortical surface segmented from the pre-operative MRI.

An intra-operative picture was taken after the electrode grid was placed over the exposed brain. Intra-operative location of each electrode were recorded with the neuro-navigation system (Medtronic, Inc., Minneapolis, MN, USA). Image guided localization of the electrode location were performed as soon as possible after the craniotomy was completed in order to minimize potential brain shift. The electrode location was recorded using the intra-operative Medtronic neuronavigation system (**Figure 2**). The accuracy of the neuronavigation approach used is well established and represents the standard method for image-guided resection of brain tumors (14, 15). The electrodes were classified as overlying the tumoral, peritumoral or macroscopically healthy tissue. This was performed for each patient according to the radiological defined boundaries as per the conventional structural MRI: FLAIR for non-enhancing diffuse low-grade gliomas and the T1 MPRAGE with contrast for contrast enhancing tumors (16) (**Figures 2** and **S2**). The peritumoral tissue was defined as an area within a two cm margin from tumor borders according to the RANO criteria (16). All MR brain images were acquired one or two days preoperatively in the diagnostic room of an iMRI-integrated neurosurgical suite using a 3T Siemens MRI scanner (Siemens MAGNETOM Verio 3.0 T, Germany) with a standard 32-channel head coil. The cortical surface was segmented from the pre-operative MRI using the Analysis of Functional NeuroImages software (http://afni.nimh.nih.gov/afni). Intra-operative photograph was superposed on the segmented cortical surface in order to confirm the electrode location in respect to the tumor with the help of visible sulcal patterns on the cortical surface obtained from the pre-op MRI (**Figure 3**). A total of 351 ECoG channels from 12 subjects were recorded for this study. The total channels per tissue type were as follows: 112 macroscopically healthy, 106 peritumoral, and 133 tumoral electrodes.

### ECoG Signal Analysis

ECoG signals were manually inspected for quality using EEGLAB for full-length data visualization. Data for electrodes, or channels, were deemed to be of untrustworthy quality if obvious artifacts were present in at least ¼ of the full length of channel data. The entire set of data for these bad channels was removed from further analysis. We calculated the power law exponent (PLE) using an in-house MATLAB script as per previous publications (17–21) using an uninterrupted 5 minutes resting-state signal. Coarse-graining spectral analysis (CGSA) (22) was applied to the uninterrupted 5 min resting-state data to separate harmonic activity from the non-harmonic or scale-free activity of interest. The power spectral density of the uninterrupted 5 min resting-state data was then calculated using Welch’s averaged, modified periodogram method of spectral estimation (23) with 50% windowed segment overlap, eight window segments, and a Hamming window type. Subsequently, power spectrums of each channel were then averaged for each tissue type and this averaged spectrum was log-log transformed according to previous studies (19, 24, 25). A notch filter was applied in software to the 45–55 Hz band to eliminate mains electricity noise, and for PLE-fitting continuity a linear interpolation replaced the spectrum across this bandwidth. MATLAB’s fit function was then used to fit a linear polynomial curve to the log-log spectrum, and the slope of this line was extracted as the PLE value. One PLE value was extracted for each tissue type, i.e., tumoral, peritumoral and respectively tumoral, and all PLEs were compared using a paired T-test. A p value < 0.05 was considered statistically significant.

## Results

**Figure 4:**
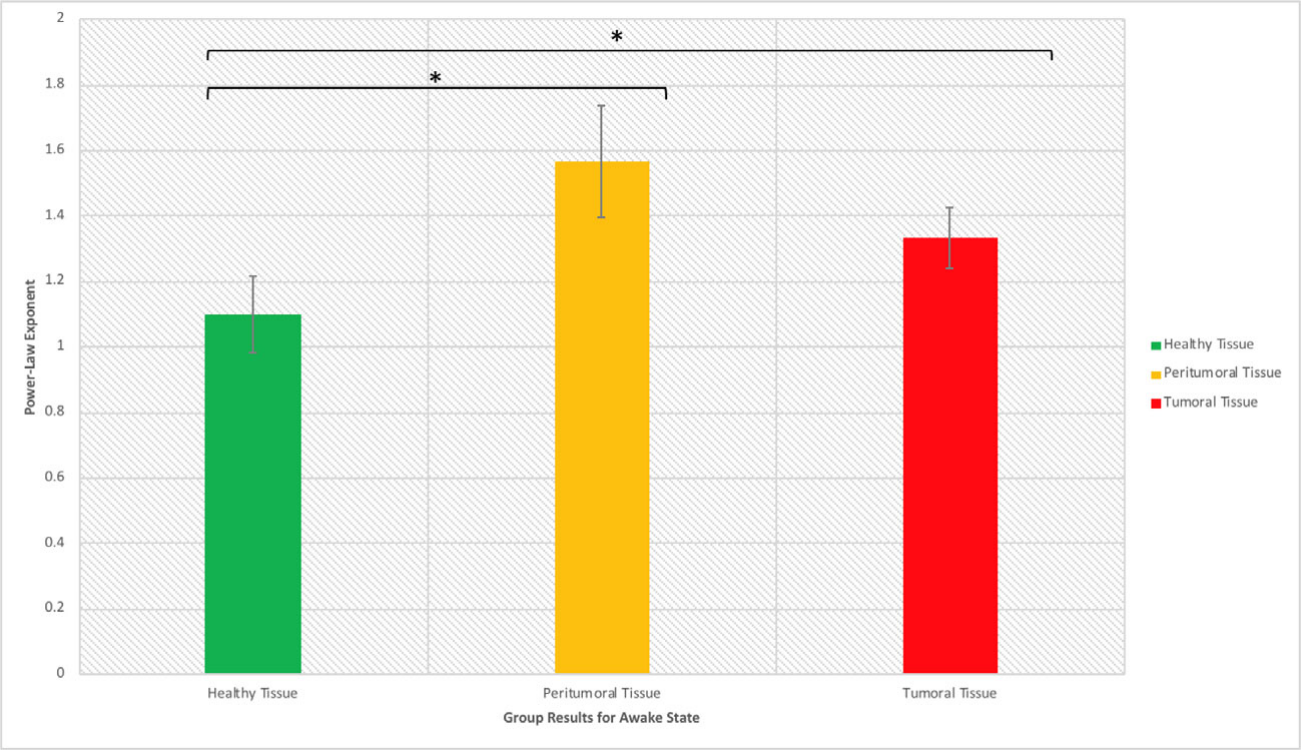
Power law exponent in the awake resting-state activity for distinguishing healthy, peritumoral and tumoral tissue (*significant at p < 0.05).

**Figure 5:**
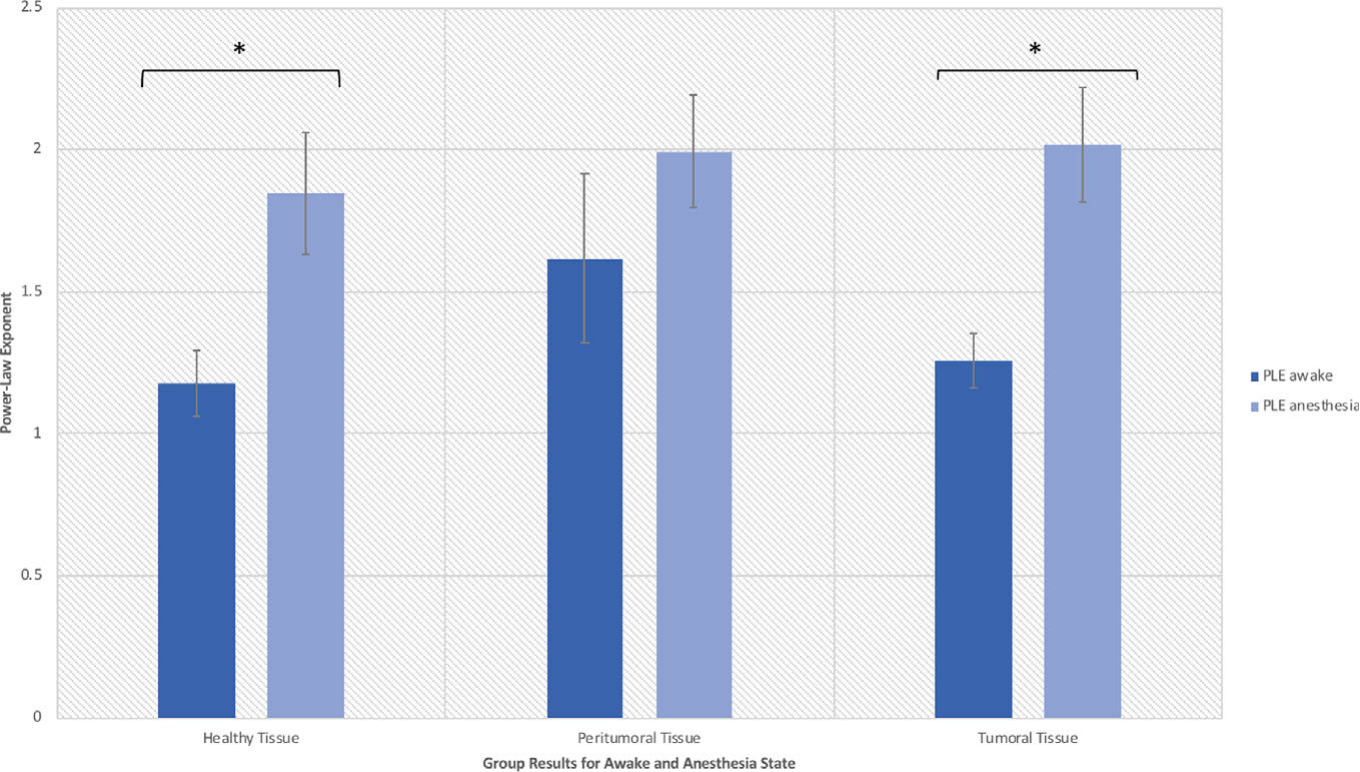
Power law exponent results in the anesthetic and awake states (*significant at p < 0.05).

When assessing scale-free measures, we noticed that the electrodes over the tumoral tissue were found to have a higher PLE value than the electrodes over the macroscopically healthy tissue for the twelve patients (**Figure 4**). Furthermore, the peritumoral tissue displayed significantly higher values than the macroscopically normal tissue (p = 0.02), which we hypothesized is due to the different nature of that tissue. The difference between PT and TT did not reach statistical significance (p = 0.10). The higher PLE values observed in the peritumoral tissue was in concordance with the observed lack of reaction to anesthesia in the peritumoral tissue (**Figure 5**), which we hypothesized that it is related to a different tissue type which maintains the already high PLE values. This can entail that the peritumoral tissue displays a higher level of entropy than the surrounding tissue. When comparing the PLE results according to the presenting symptoms (respectively with or without seizures) or according to previous antiepileptic drug use, no significant differences were observed (**Figures S3–S5**).

**Figure 6:**
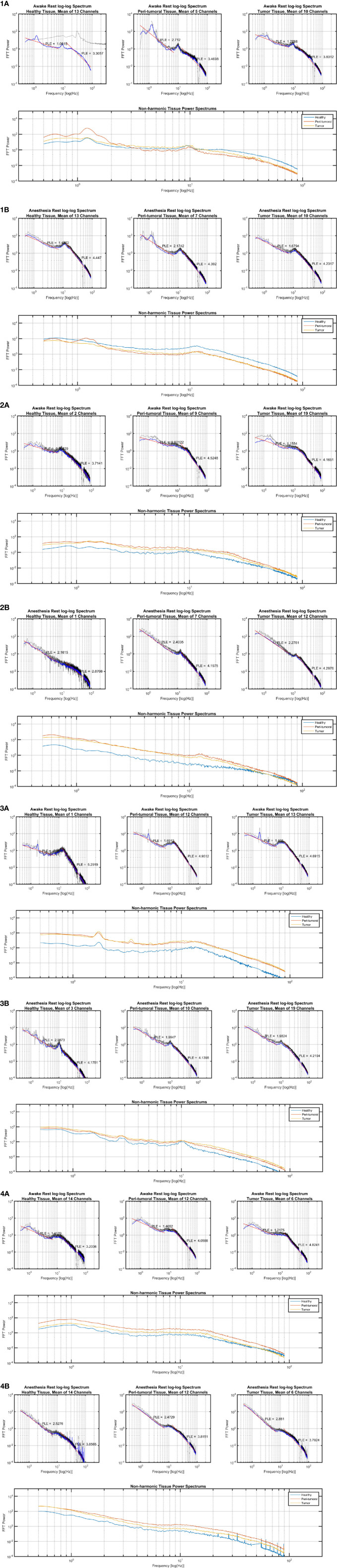
Power law exponent results in individual cases. **(1A)** Subject 1 in awake state, **(1B)** Subject 1 in anesthetic state; **(2A)** Subject 2 in awake state, **(2B)** Subject 2 in anesthetic state; **(3A)** Subject 3 in awake state, **(3B)** Subject 3 in anesthetic state; **(4A)** Subject 4 in awake state, **(4B)** Subject 4 in anesthetic state.

For the six patients presenting with multiple seizures for which ECoG recordings were obtained in the two anesthetic states (i.e., awake and anesthesia), we observed that the electrodes overlying the peritumoral tissue displayed smaller changes between the anesthesia and awake state than the changes observed across the adjacent tissues, respectively over the TT and macroscopically HT (**Figure 5**). Furthermore, we observed distinct features of the PT’s power spectrum when comparing with the power spectrum of the HT and TT as showed in the individual results (**Figure 6**). For instance, patient #1 displayed a higher power spectrum of the peritumoral tissue in the lower frequencies in respect to the healthy and tumoral tissue. Nonetheless, the peritumoral tissue shows the opposite pattern in the higher frequencies of the power spectrum; the patient had a WHO Grade III tumor. Patients #2 and #3 showed a different pattern where the power spectrum of the peritumoral tissue was similar to that of the tumoral tissue; those patients had a grade III and, respectively, grade IV tumor. The three patients with a WHO grade II tumor showed an intermediate PT power spectrum (patient #5) and respectively a higher peritumoral power spectrum than the other tissues (patient # 4 and # 6). The same power spectrum pattern remained in the anesthetic state. Further genetic analysis would be required to assess whether there is any correlation between the tissue infiltration and the different electrophysiological features.

In addition, given that the glial tumor can displace otherwise healthy non-glioma neural tissue and that this mass effect can cause electrophysiological changes in the intraoperative ECoG, we performed a subgroup analysis according to the presence of mass effect. Two subjects included in this study presented with intracranial mass effect, as identified on the preoperative MRI. The PLE of these subjects (sample size = 61, μ = 3.218, σ² = 0.179) was not found to be different from the PLE of the subjects presenting without a mass effect (sample size = 290, μ = 3.306, σ² = 0.486, P = 0.231). Based on these results, the intracranial mass effect was not considered to influence the PLE in this study. It should be noted however, that only two subjects presented with glioma-related mass effect. Also, given the limited time available for intra-operative recordings during the awake procedures, we, unfortunately, could not perform post-resection ECoG recordings.

Furthermore, the distance between tumor and the brain surface is also important and to further explore this factor we looked at the influence of the cortical/subcortical tumor location. To study the effect that glioma’s proximity to the cortex has on the PLE results, we compared the PLE of patients with gliomas predominantly involving the cortical surface with the PLE of patients having a more extensive subcortical involvement. The PLE of patients with predominant cortical involvement (sample size = 233, μ = 3.238, σ² = 0.540) was lower than the PLE of patients with both cortical and subcortical glioma (sample size = 118, μ = 3.416, σ² = 0.207, P = 0.0067, α = 0.0167). The PLE of patients with both cortical and subcortical glioma was less variant than the PLE of patients with only cortical glioma. This suggests that further analysis should focus on studying the subcortical glioma involvement, and a continuous representation of glioma depth from the cortical surface could be incorporated in the future to study the effect that glioma depth has on the PLE of the surrounding neural tissue.

Lastly, the importance of the glioma’s molecular and histological characteristics cannot be overemphasized given the known strong correlation with clinical outcomes. Although we have a small sample of subjects, in order to study the effect that this mechanism could have on the PLE results, all channels for each subject were labeled as low-grade (grade II) or high-grade (grade III & IV) which coincided with an IDH-mutant and respectively an IDH wild-type status. The PLE of low-grade glioma-associated channels (sample size = 236, μ = 3.266, σ² = 0.505) was not found to be different from the PLE of high-grade associated channels (sample size = 115, μ = 3.348, σ² = 0.318, P = 0.248). From this analysis, it was concluded that there was not enough evidence to suggest that an unbalanced representation of glioma graded tissue in this study would bias the PLE results presented.

In summary, ECoG recordings performed prior to glioma resection allowed us to investigate scale-free related changes in the resting state activity of tumor tissue and its reactivity to anesthesia. We observed an increased PLE in the anesthetic state for all types of tissues (with a larger extent within the tumoral tissue) and additionally, a different behavior of the PT when comparing PLE in the two anesthetic states (smaller PLE changes than in the adjacent tissues). We thus noticed a lack of reaction to anesthesia in the peritumoral tissue and hypothesized that it is related to a different tissue type which maintain the already high PLE values.

## Discussion

Extrapolating our group’s previous work in functional MRI, for the first time to our knowledge, the analysis of the signal from ECoG electrodes in this study concentrated on power law exponent. Power laws have been reported in many distinct scientific, geographical and social phenomena and have been associated with long memory behavior, self-similarity and fractal structures (26). Power law was initially utilized to characterize the distribution of wealth in a society being known as the Pareto principle, where a small percentage of the population has a large proportion of wealth. Similarly, our investigation of the cortical electrophysiological changes occurring in the setting of twelve patients undergoing craniotomy for resection of glioma revealed distinct electrophysiological features across the tumoral area compared to the surrounding tissues, namely a higher PLE value over the tumoral tissue than for the surrounding areas. The tumoral tissue PLE difference between the two anesthetic states resembled to the one observed in macroscopically healthy tissue which could imply a functionally independent state of the tumoral tissue. This has been previously suggested in recent articles, which demonstrated that gliomas develop functional multicellular network structures where tumor cells “communicate and cooperate with each other in a complex but ordered manner that is by itself reminiscent of a functional organ” (27, 28). Therefore, as a correlate, the tumor could be seen like the tall trees that continue to grow using more resources from the ecosystem on the expense of smaller trees (29), for instance surrounding cells, that do not have access anymore to the scarce resources.

Furthermore, the tumor’s surrounding tissue showed specific features. A universal definition of the peritumoral area in glioma is lacking and its complex features are not yet elucidated (30, 31). Nonetheless, current guidelines recommend the radiation therapy target volume to be delineated on the surgical cavity with the addition of a 20-mm margin, which represents the most common site of recurrence (30–32). Gliomas are well known to infiltrate the brain tissue well beyond the radiological boundaries of the tumor (33, 34) and thus, our HT electrodes should be perceived as macroscopically healthy tissue. For instance, in **Figures 5** and **6**, the PT tissue displays an intermediate feature between the TT or macroscopically HT-electrodes as well as a smaller PLE change when investigating the difference between the two anesthetic states. We suspect that these features are related to tumor cell infiltration. Further studies with prolonged recordings and combined genetic analysis could test our hypothesis. Also, the loss of PLE change in the PT between the awake and anesthesia states in patients presenting with seizures could suggest a distinctive state of the peritumoral area. As such, PT could represent an interlaid area between the brain and the functional tumor with specific characteristics favoring the generation and propagation of seizures. Being characterized as an interface between the tumoral and healthy brain, the peritumoral tissue could thus represent an area requiring a higher energy expenditure as it represents the invasion front of tumor cells into the neighboring tissue (33). Hatcher et al. recently reported spontaneous episodes of cortical spreading depolarization that arose frequently from the peritumoral region (35). This depolarizing phenomenon occurs in the setting of a variety of pathological states and represents a spreading loss of ion homeostasis, altered vascular response while in healthy tissue an increased electrical activity is coupled with the release of vasodilatory factors such as nitric oxide to increase local blood flow to meet increased energy expenditure (36). Although in GBM, the peritumoral tissue was found to contain cancer stem cells, Cubillos et al. reported that the concentration of taurine, an amino acid that may have a protective effect or be involved in cell proliferation, was found to be higher in the peritumoral tissue of gliomas (37). The large histological and molecular heterogeneity of gliomas highlights the need for a better understanding of the underlying biomolecular characterization and biochemical reorganization occurring in this important region. This could give more insights in terms of the resulting electrophysiological changes recorded over these regions in order to allow the development of personalized therapies.

The origins and mechanisms of GRE are multifactorial (3) and the current evidence suggests that there is a link between epileptogenesis and glioma cell infiltration in the peritumoral area since epileptic activities seem to arise from the neocortex surrounding the gliomas (31–33). Also, glioma cell infiltration seems to promote growth and recurrence of tumors at sites around their core (34, 35). In a recent study performed by Pallud et al., it was demonstrated that in slices of human tissue, the peritumoral neocortex infiltrated by glioma cells generates spontaneous interictal discharges that depend on both glutamatergic and GABAergic signaling (36). This link may explain both the anti-epileptic effects of oncological treatments (38, 39) and the increase in seizure frequency as tumors progress (9, 40, 41). Early operative intervention and gross-total resection contributes strongly to seizure freedom and improving quality-of-life (1, 42, 43). Emerging evidence advocates an aggressive and early surgical approach and suggests that the opportune time to perform epilepsy surgery may be when the patient is already undergoing oncosurgery in the same area (10). As such, proper seizure diagnosis and therapy requires accurate identification and management of the infiltrated peritumoral tissue.

Electrocorticography plays an important role in the localization of epileptogenic foci and evaluation of the effects of microsurgical procedures intraoperatively. An accurate estimation of the epileptogenic cortex and its removal requires the estimation of spatial spread of ECoG. Recent studies estimating the spatial spread of ECoG suggested that although brains’ signals capture different features of the neural network, ECoG records a local signal (diameter of ∼3 mm) confirming the precision required for its use in determining the epileptogenic focus (44). Furthermore, Berger et al. previously proposed that electrocorticography-guided epilepsy surgery for pediatric patients with brain tumors is highly effective (45). Given that some patient populations cannot participate during awake craniotomies (for example with severe neurological deficits), several investigators have utilized high-gamma ECoG changes during language and motor tasks to localize eloquent cortex and have reported good concordance with cortical stimulation mapping (46–50). High gamma activity (HGA) between 80 and 140 Hz on electrocorticography is assumed to reflect localized cortical processing, whereas the cortico-cortical evoked potential (CCEP) can reflect bidirectional responses evoked by monophasic pulse stimuli to the language cortices when there is no patient cooperation. The use of “passive” mapping was thus proposed by combining HGA mapping and CCEP recording without active tasks during conscious resections of brain tumors (51). In terms of localizing the ECoG electrodes in relation to the tumor, an approach using solely the intra-operative photography might be prone to errors given that areas invaded by the tumor might not be clearly delineated and the sulcal patterns may not be easily segmented. However, as the neuronavigation is used clinically to decide the extent of resection, we confirmed the electrode locations using the neuronavigation probe thus reducing the risk of error in electrode localization. Furthermore, the ECoG recording was performed as soon as the craniotomy was completed in order to minimize the effect of the brain shift.

In tumoral epilepsy surgery, the first aim is to maximize the extent of tumor resection while minimizing postsurgical morbidity, in order to increase the median survival as well as to preserve the quality of life (52). Gross-total resection of tumors is also a critical factor in achieving seizure freedom (40) in infiltrating tumors such as gliomas, where these lesions migrate along white matter tracts. A significantly higher resection volume was associated with a higher chance of favorable seizure outcome in multiple studies, especially in long-term epilepsy-associated with glial tumors (53). Extraoperative mapping by strips/grids is often not sufficient in tumoral surgery, since in essence, it allows the study of the cortex but cannot map subcortical pathways (52). Although, recent research demonstrated that awake craniotomy can be performed safely without ECoG, even in patients with preoperative intractable epilepsy (54), some studies showed that when complete ECoG-guided resection is performed there is a greater likelihood of long-term seizure freedom (55). Awake functional mapping allows surgeons to maximize tumor resection while minimizing neurological deficits, thus improving patient’s survival and quality of life (56, 57). This approach has allowed an improvement of both oncologic and neurological outcomes however, many patients do not reach the complete postsurgical control of seizures. Therefore, the use of more precise preoperative evaluation and adequate surgical techniques is desirable for those patients. Recently, Ius et al. outlined the importance of the EOR on postoperative seizure outcome as the EOR was independent of the molecular class of the tumor (58). Similarly, Xu et al. showed that an EOR threshold of more than 80% was associated with a long-term seizure freedom (59). In a larger multicenter investigation, Still and colleagues demonstrated that for patients with a supratentorial DLGG, postoperative seizure control was more likely to occur when the EOR was ≥ 91% and/or when the residual tumor volume was ≤ 19 cc (60). Our findings of progressive PLE changes from the tumor core could be used to further delineate the electrophysiological changes induced by the tumor (**Figure S6**). However, these preliminary findings need to be first correlated with genetic/histological data. Future prospective studies integrating direct electrical stimulation and ECoG could refine the supramarginal resection technique when functionally possible. A multidisciplinary approach including epileptologists for a comprehensive assessment and accurate localization of the epileptogenic focus are primordial for a better understanding of the potential clinical applications of our findings. The correlation of the intra-operative electrophysiologic changes with clinical markers and outcome measures is an important aspect that should be explored in the future with well-designed prospective studies.

The concept of individualized surgery for managing glial tumors is based on the goal of achieving a maximal tumor resection and seizure freedom without inducing new neurological deficits (61–64). We already dispose of ample information in terms of the clinical and biological behavior of gliomas. Nonetheless, the persistent challenge remains integrating the information obtained from various analyses in a way that provides a comprehensive view of the alterations underlying the interplay between epileptogenesis and oncology. Scale-free measures such as PLE are ubiquitously present in many scientific topics and this research represents the first confirmation of its relevance to the tumoral tissue. Further research including in-vivo neural recording could help improve our insight into latent sources of PLE changes.

## Conclusion

In summary, we present for the first time distinct power law exponent features in the tumoral and peritumoral tissue of gliomas that seems to represent a critical structure both for the onset and propagation of epileptiform activity and for infiltration by glioma cells. Although neuro-oncology and epilepsy surgery have traditionally been separate disciplines, we postulate that the analysis of the mechanisms underlying the tumor related epilepsy might equally yield significant insight into tumorigenesis. We expect that the contributions made from a multidisciplinary approach to unravel the complex pathways underlying the clinical features of gliomas related epilepsy will lead to meaningful progress for the patients affected by these devastating tumors.

## Data Availability Statement

The raw data supporting the conclusions of this article will be made available by the authors, without undue reservation.

## Ethics Statement

The studies involving human participants were reviewed and approved by the Huashan Hospital ethics board (approval nos. KY201209 and KY2015-168). The patients/participants provided their written informed consent to participate in this study.

## Author Contributions

Conception and design: GN, J-SW, JL, and DG. Acquisition of data: DG, JL, and J-SW. Analysis and interpretation of data: BL, DG, J-SW, NJ, JL, GN, ET, and AS. Drafting the article: DG. Critically revising the article: J-SW, DG, NJ, ET, AS, and GN. Reviewed submitted version of manuscript: all authors. Approved the final version of the manuscript on behalf of all authors: GN. Statistical analysis: BL, DG, and JL. Administrative/technical/material support: J-SW and GN. Study supervision: J-SW and GN. All authors contributed to the article and approved the submitted version.

## Funding

This study was supported by The National Key Technology R&D Program of China (grant no. 2014BAI04B05 [J-SW]), and Shanghai Shenkang Hospital Development Center (grant no. SHDC12018114 [J-SW]).

## Conflict of Interest

The authors declare that the research was conducted in the absence of any commercial or financial relationships that could be construed as a potential conflict of interest.
