# Supplementary material for: Scale-Free Analysis of Intraoperative ECoG During Awake Craniotomy for Glioma
Source: Front Oncol. 2021 Feb 23;10:625474. doi: 10.3389/fonc.2020.625474 (PMC7942167; doi:10.3389/fonc.2020.625474)
Supplement: Supplementary file 1 [file DataSheet_1.docx]

**Figure S1.** Power Law Exponent results in individual cases for remaining eight patients.

Patient 5 – awake state


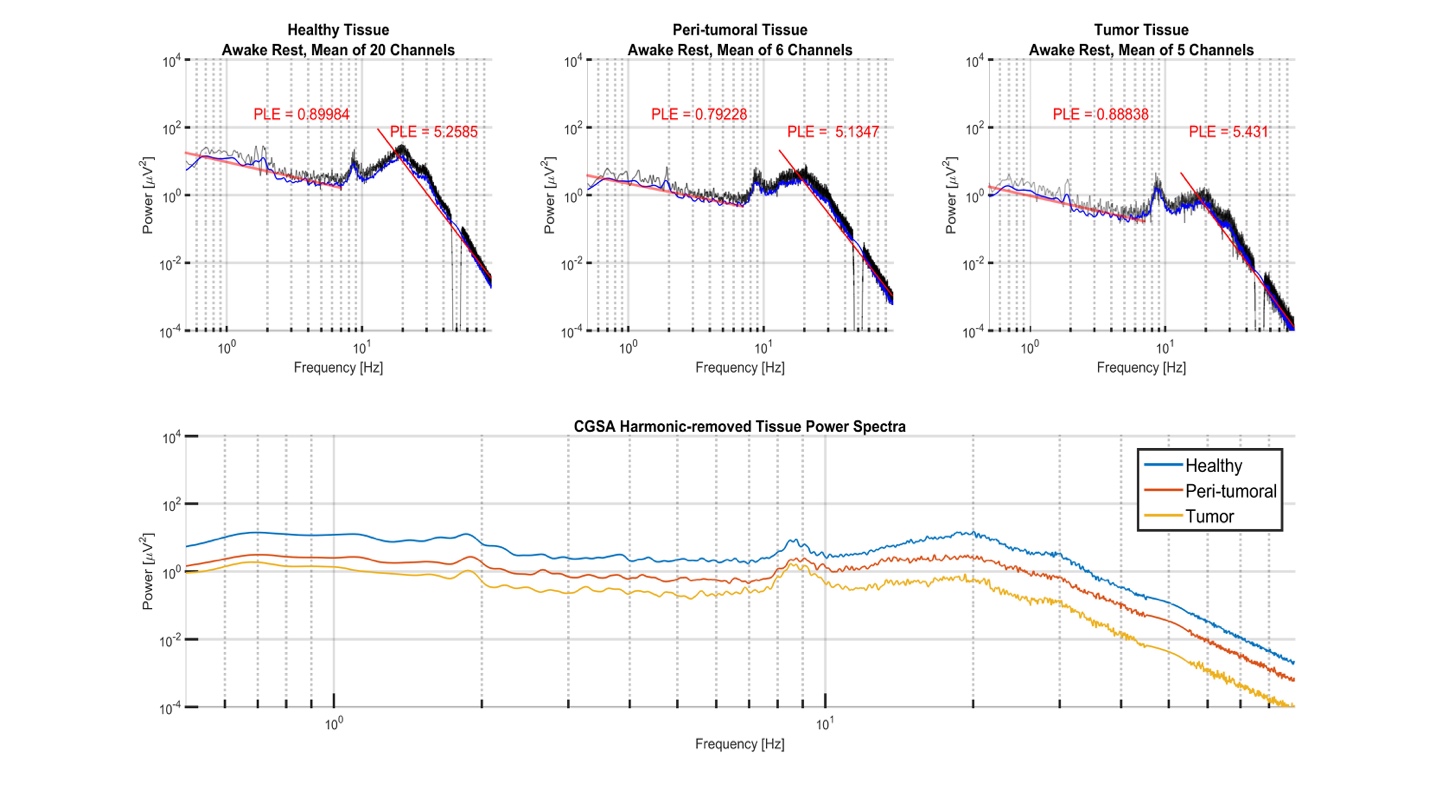


Patient 5 – anesthesia state


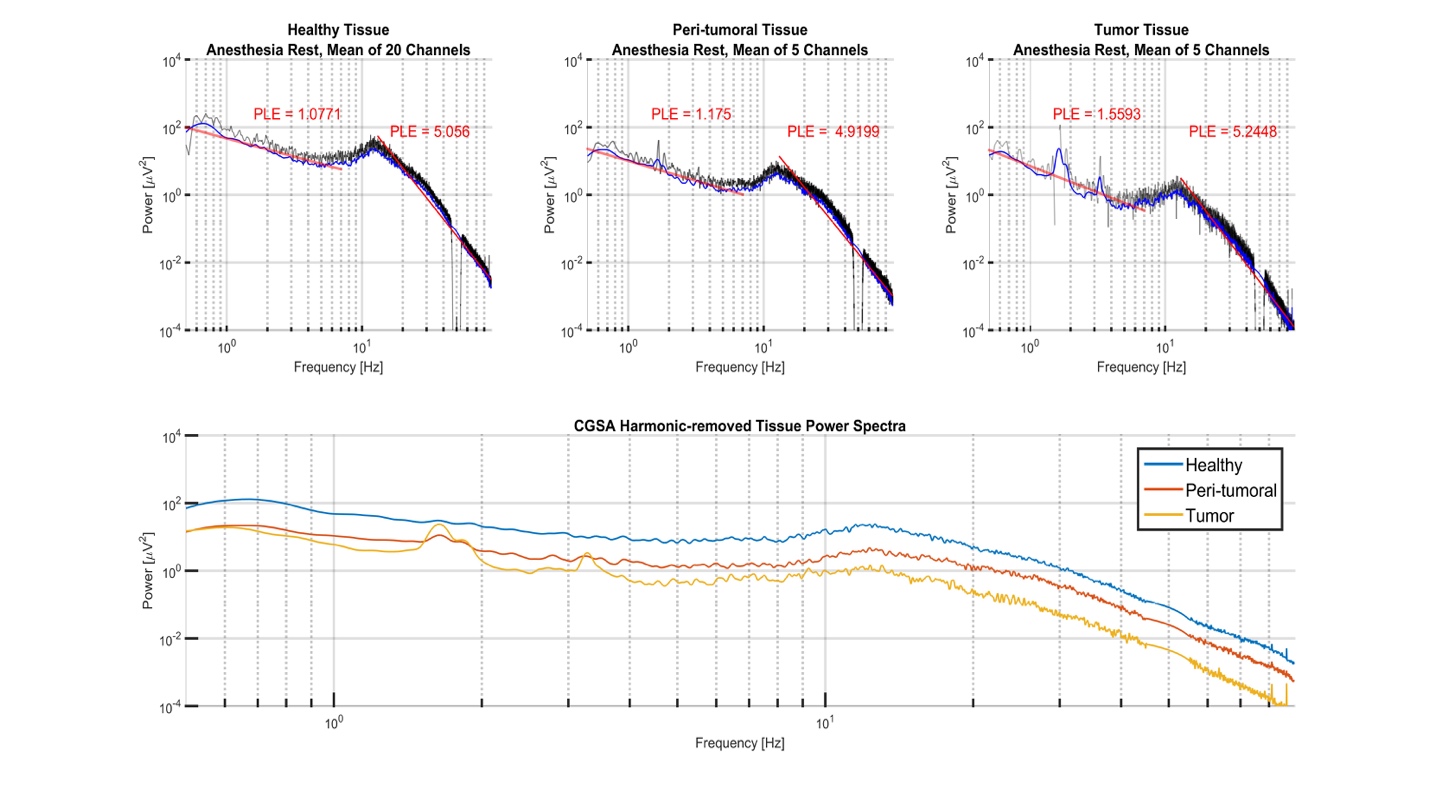


Patient 6 – awake state


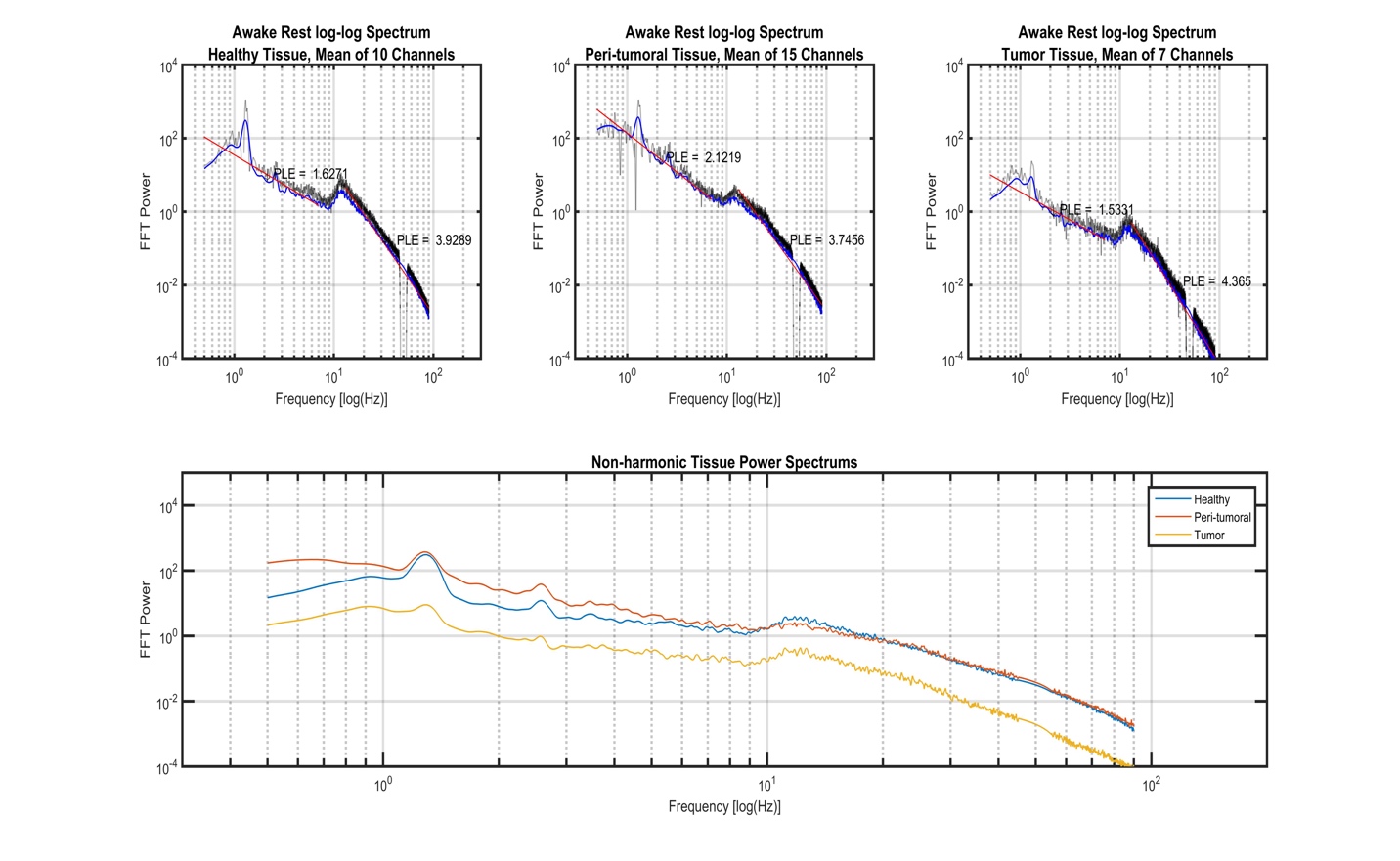


Patient 6 – anesthesia state


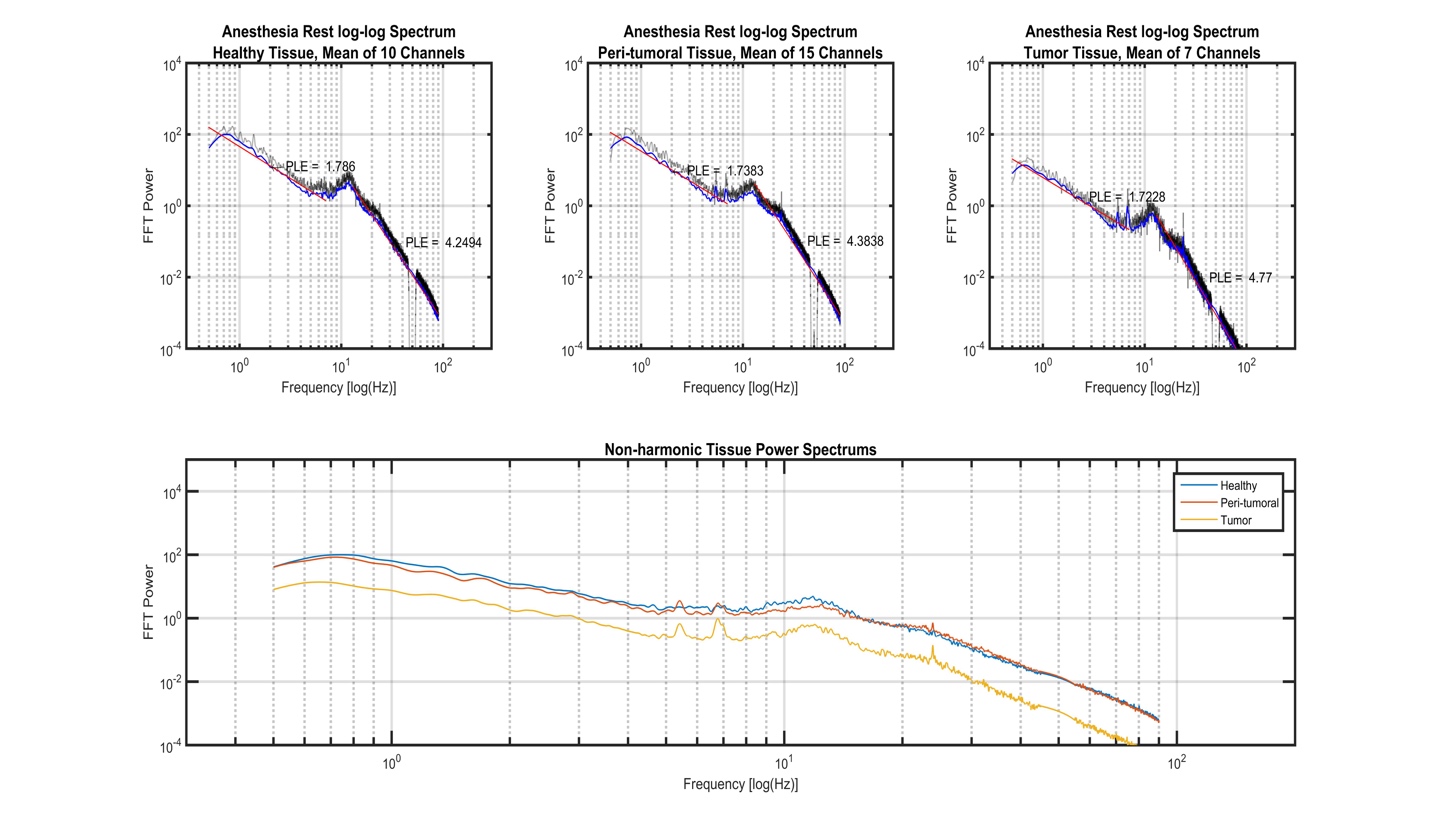


**B**. Patient 7


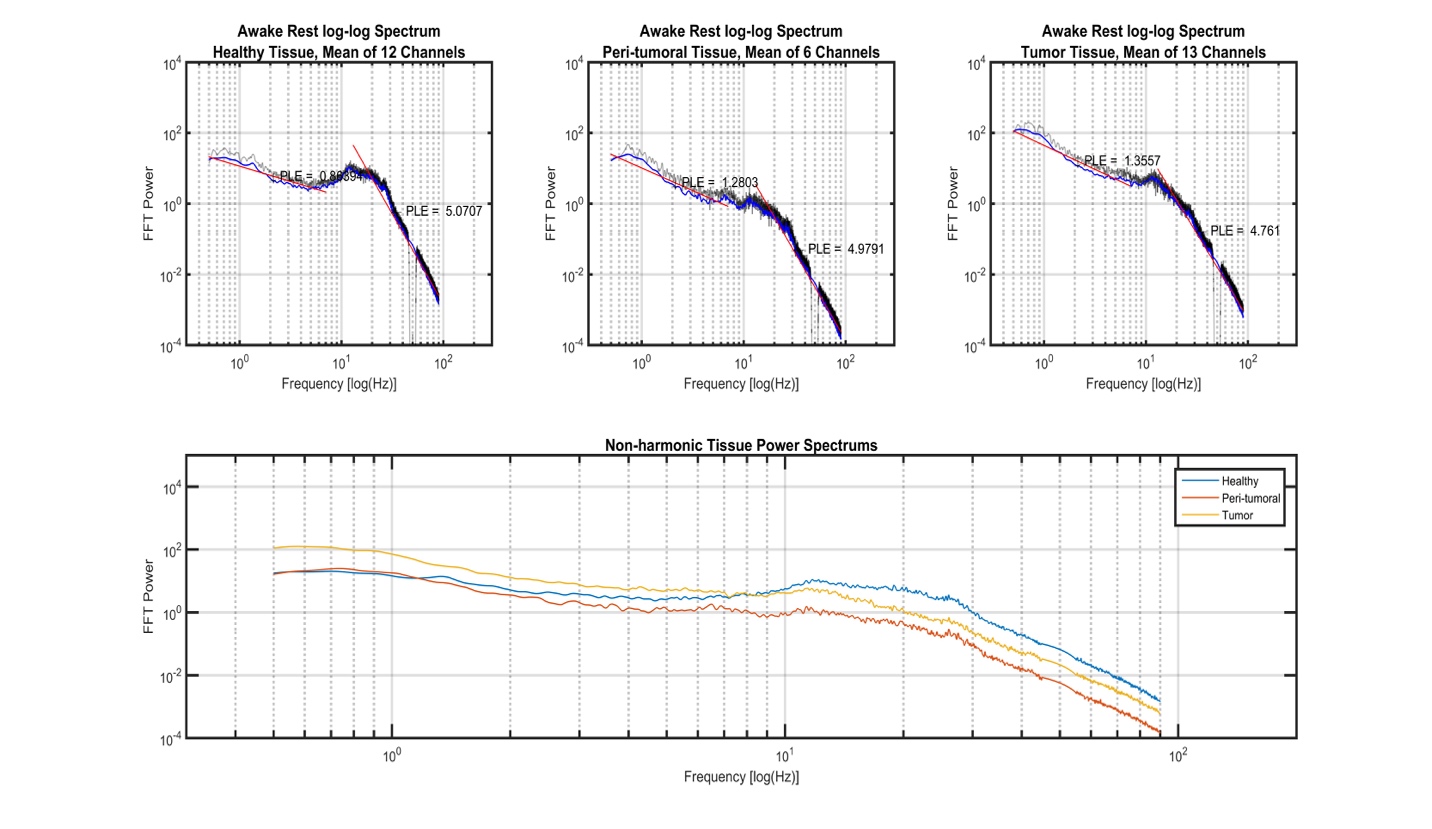


**C**. Patient 8


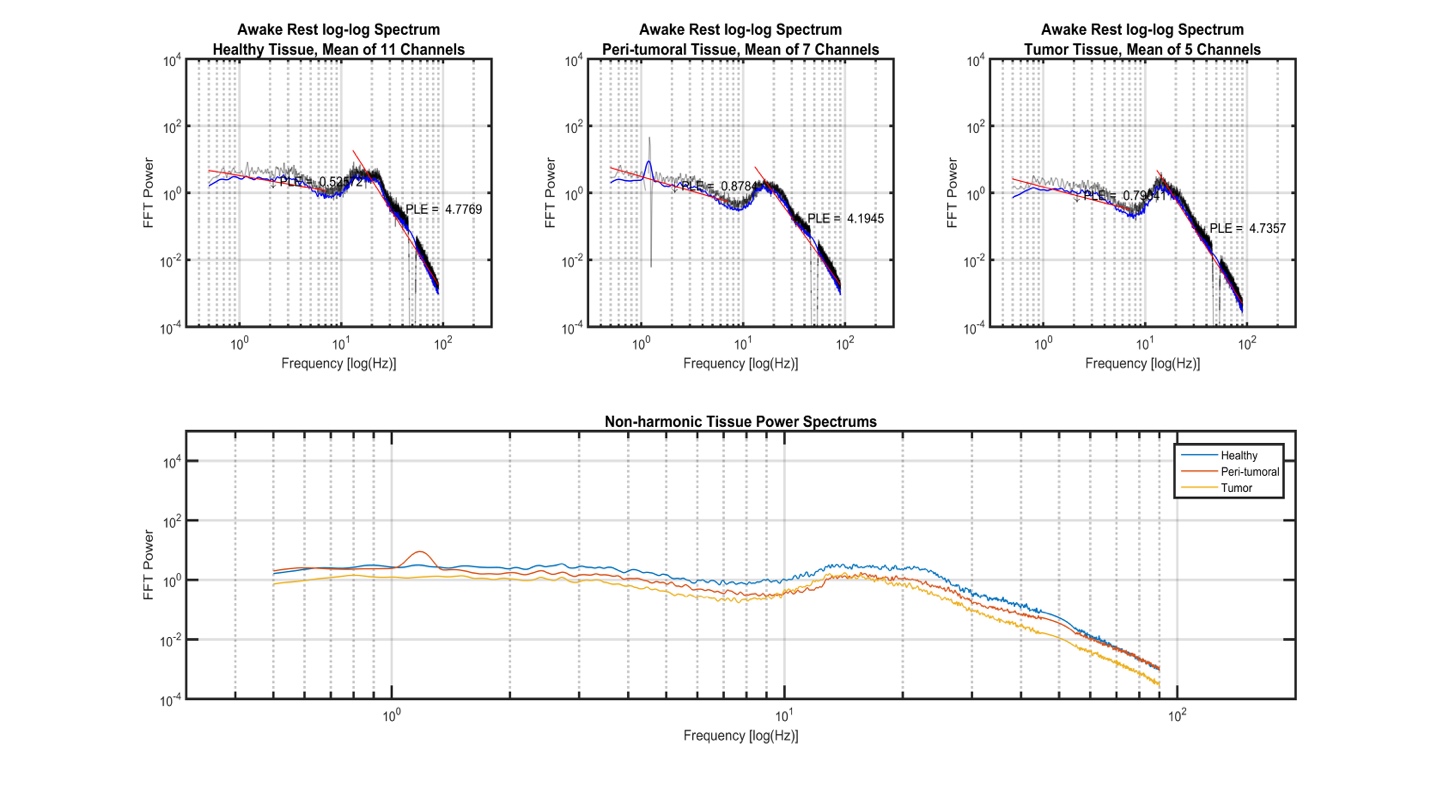


**D**. Patient 9


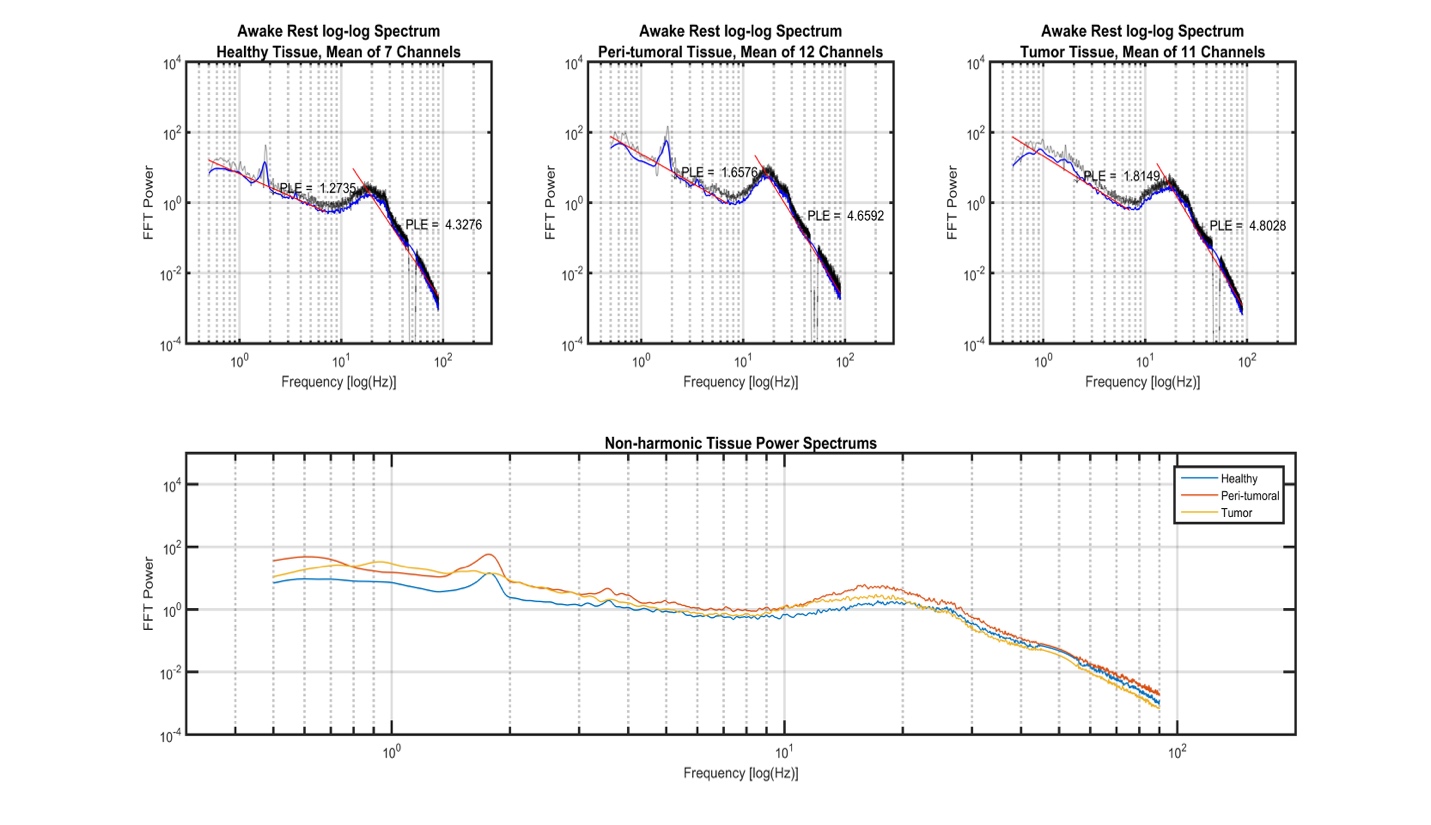


**E.** Patient 10


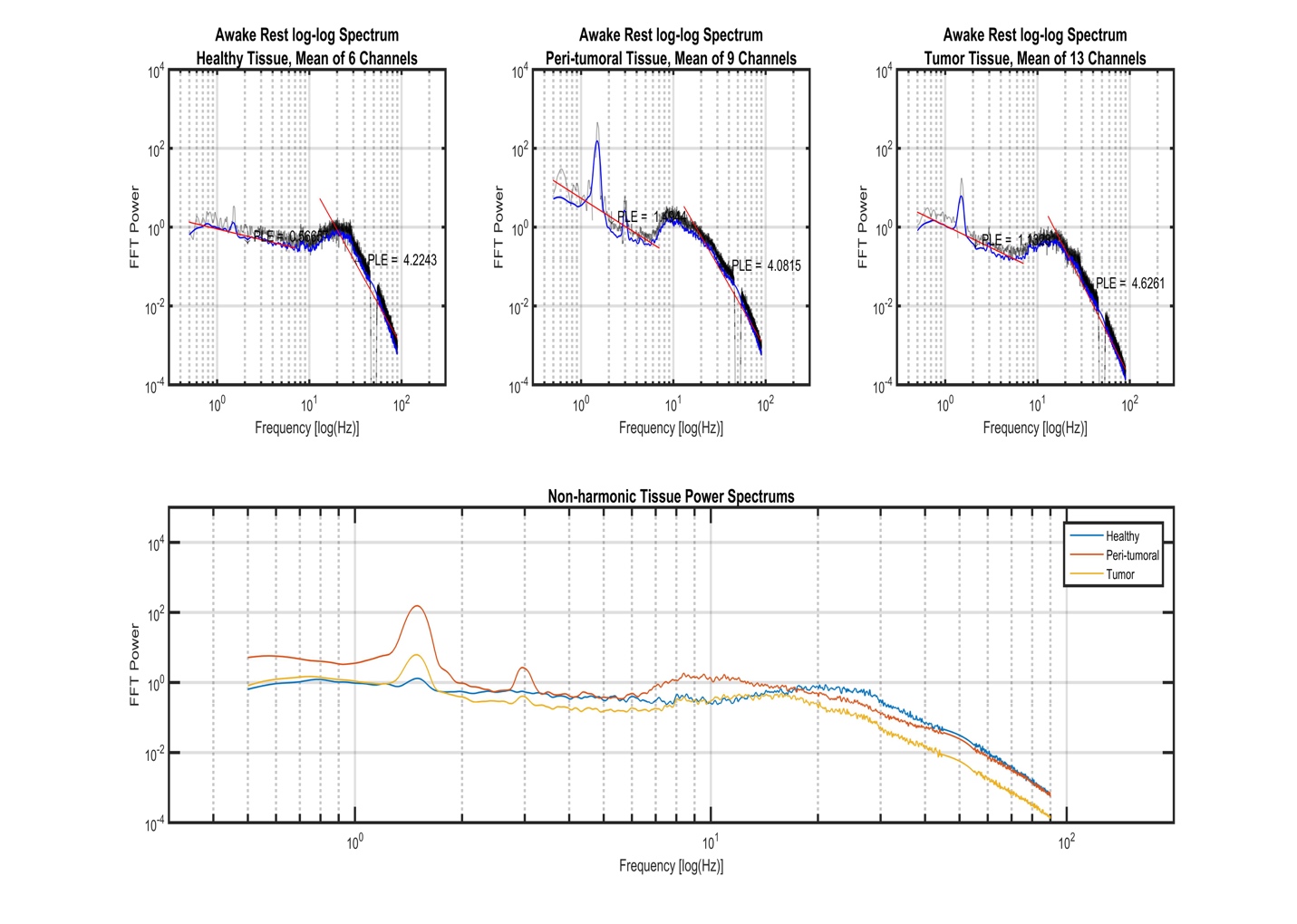


**F**. Patient 11


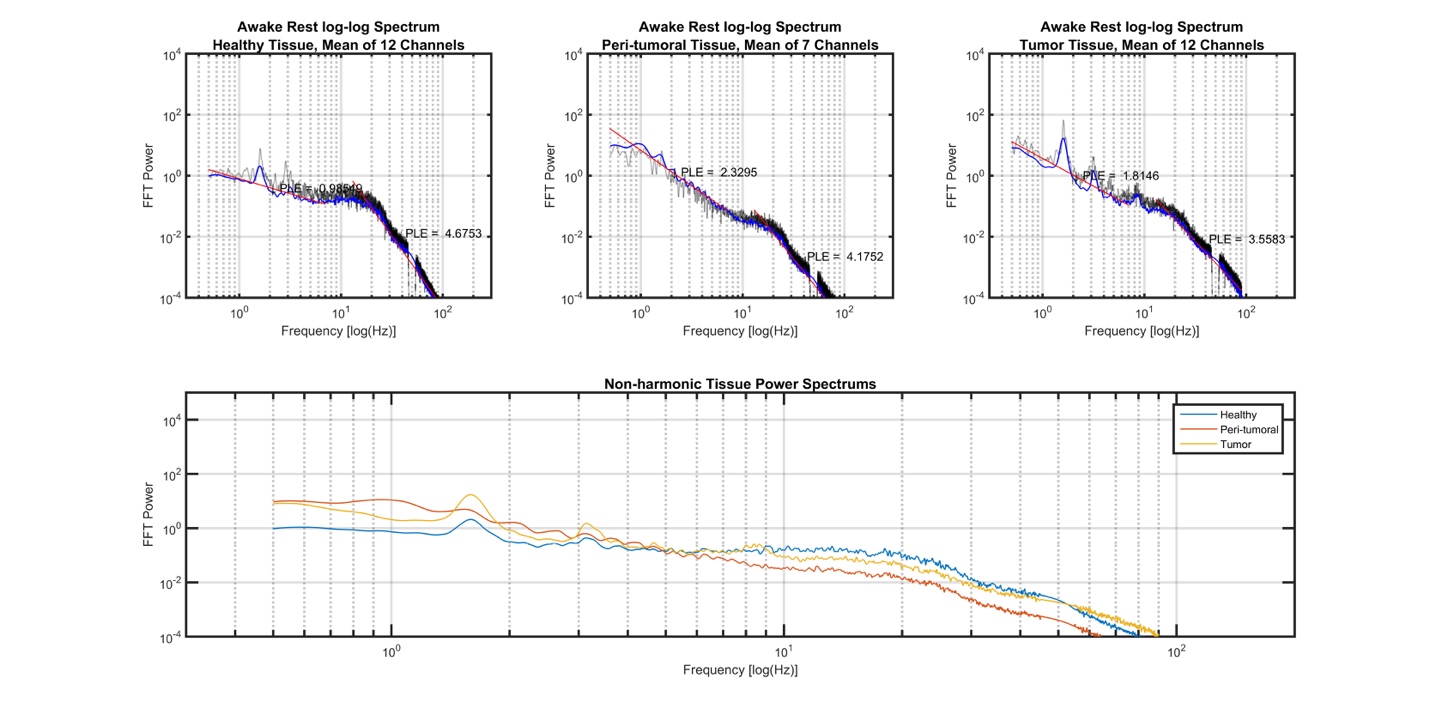


**G.** Patient 12


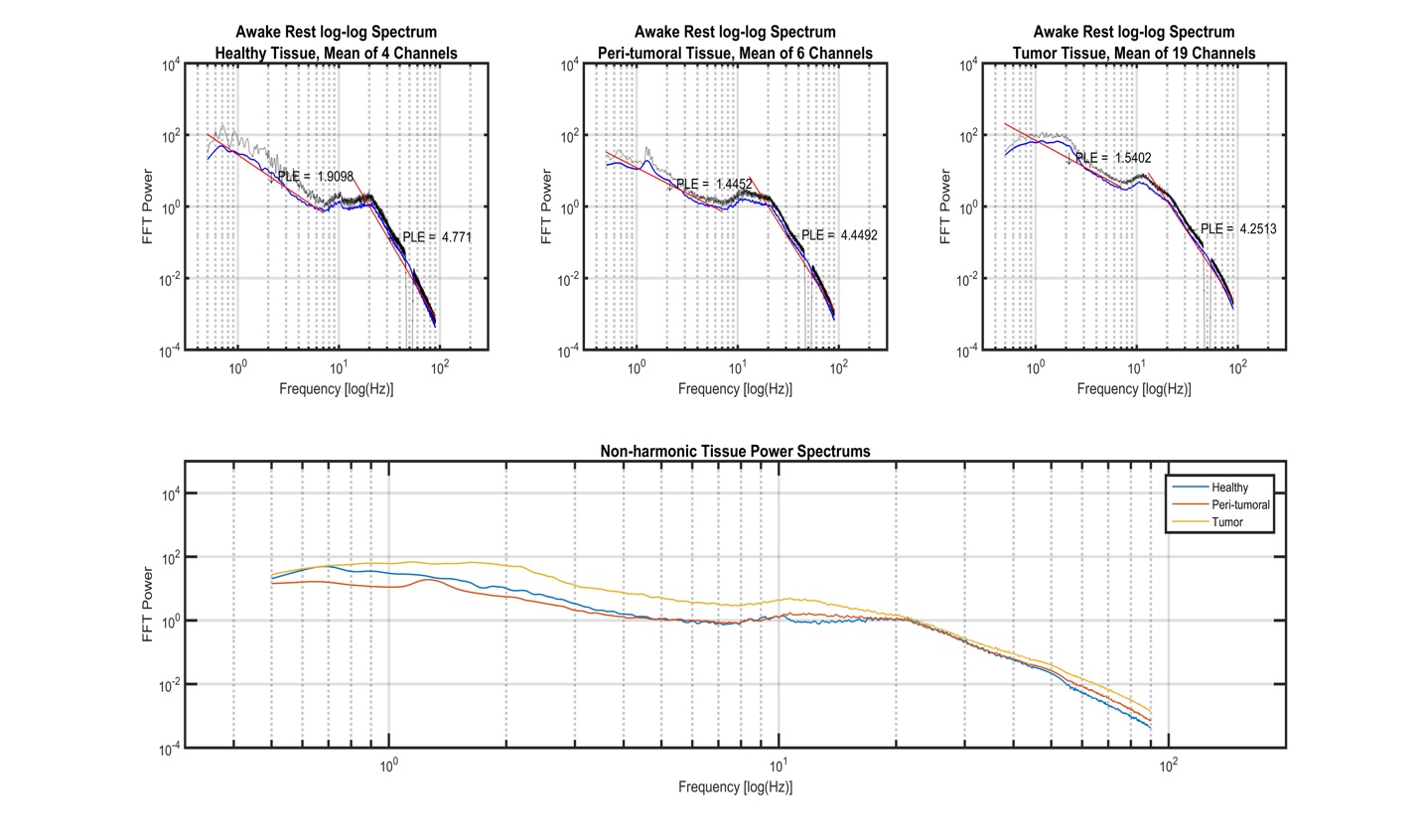


**Figure S2.** Pre-operative Magnetic Resonance Imaging and 3D representation of the tumor volume for each individual patient.


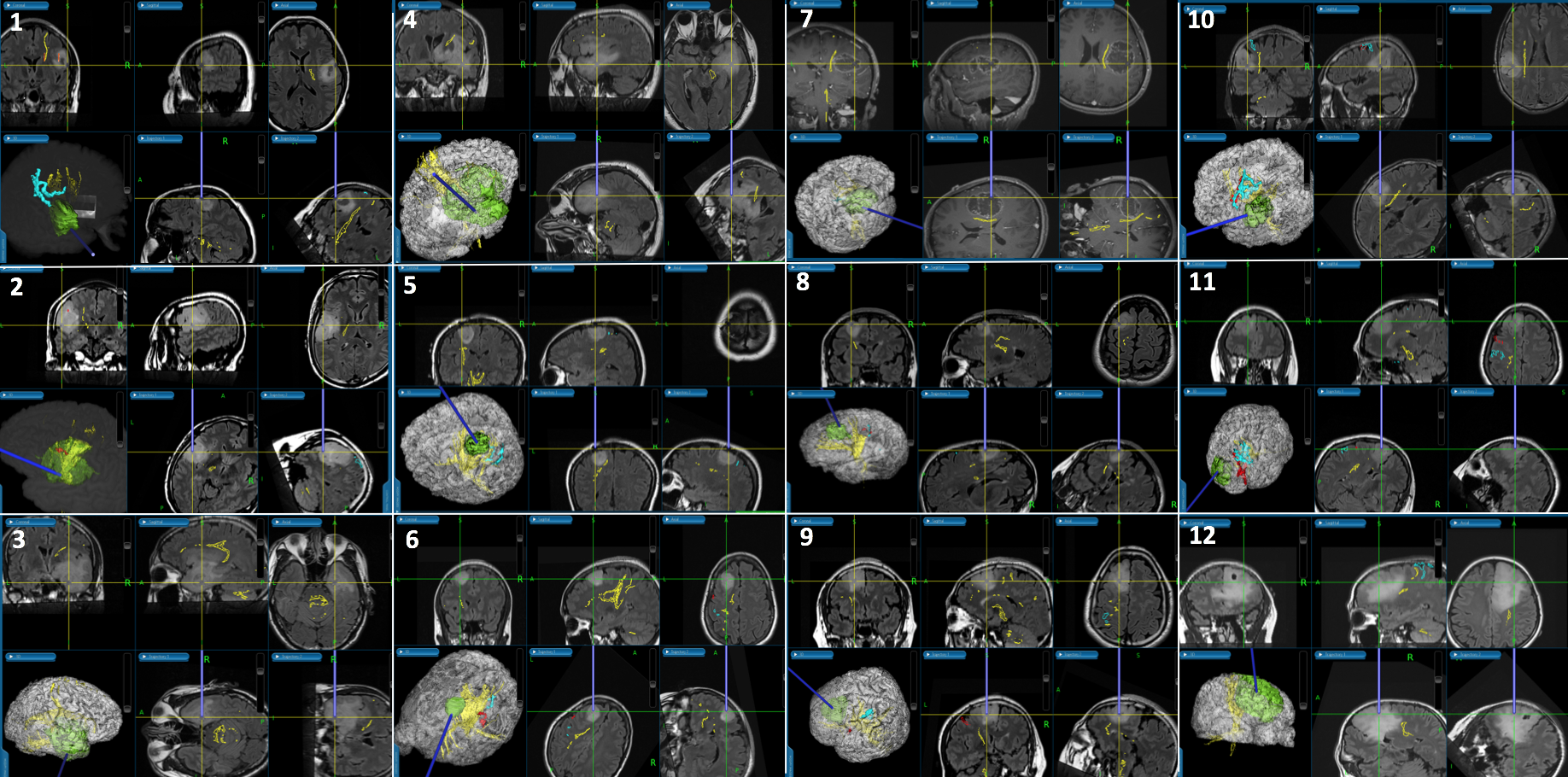


**Figure S3.** Group results according to symptoms (seizures versus no seizures) at presentation in the three tissue types (HT, PT and TT). P>0.05


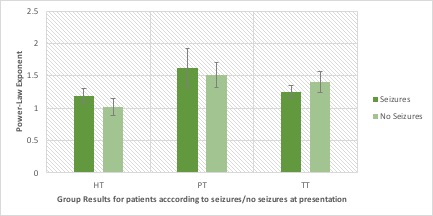


**Figure S4.** Group results according to antiepileptic drug use in the awake state for the three tissue types (HT, PT and TT). P>0.05


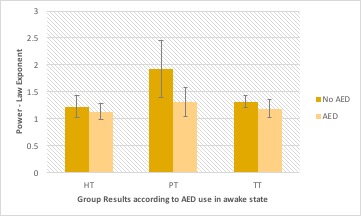


**Figure S5.** Group results according to antiepileptic drug use in the anesthetic state for the three tissue types (HT, PT and TT). P>0.05


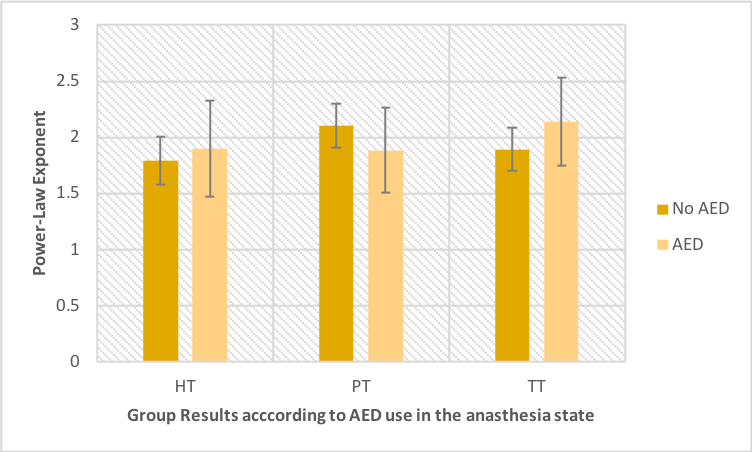


**Figure S6.** PLE surface plot for one representative patient shows interpolated values between the 4x8 PLE matrix of one PLE value per electrode; the resulting interpolated matrix is sized 16x32.
